# Early Diagnosis of Gallstone Disease1Abstract of oration on Surgery, before the West Virginia Medical Association, at Elkins, October, 1909.

**Published:** 1909-12

**Authors:** John Egerton Cannaday

**Affiliations:** Charleston, W. Va


### Early Diagnosis of Gallstone Disease.[1]

By JOHN EGERTON CANNADAY, M.D., Charleston, W. Va,

IT is not my purpose to speak of the classic symptoms of gallstone disease in its final, sometimes, hopeless stages, with these we are familiar. The average textbook describes terminal stages and dead house findings to the serious detriment of the patient. There is relatively speaking no such thing as symptomless stone in the gall-bladder. If we inquire carefully into the past history, we will find the symptoms in abundance. We had as well speak of a symptomless stone in the urinary bladder as in the gall-bladder. Neither gallstone colic nor jaundice are at all necessary to the diagnosis of gallstone disease. The sooner we get over this fallacy the better it will be for our patients. The very earliest symptoms are often quite trivial in character and frequently referable to the stomach. There is a feeling of fulness, weight or distention in the epigastrium. This is both persistent and annoying. It is generally relieved by belching, and vomiting gives complete relief. Relief may be given by bending the body forward, by loosening the clothing, or by flexing the thigh or the body. There is at times a little pain in the gall-bladder region, at times a respiratory effort is cut short by a sudden stabbing pain. There may be a slight chilliness in the evening with a goose flesh feeling of the skin. These symptoms have often gone unrecognised. About every fourth woman past middle age and a few less men have gallstones.

By constant care as regards the diet, hygiene and the use of laxative mineral waters, it is often possible for these patients to keep in a condition of comparative comfort. But if we wait numerous retrograde changes may take place in the liver, gall-bladder, and pancreas. When the time for operative treatment becomes imperative, the mortality risk is high and the technic of the work difficult. The early operation is easy, safe, and prophylactic. The question of removal of the gall-bladder or its drainage is largely dependent on the practice of the individual operator as to whether he is inclined to operate early or late. The man who operates early will be able to cure most of his cases by simple drainage, while the one who waits for terminal stages, will find his pathology so serious that removal rather than repair will be often an imperative indication.

---

1. Abstract of oration on Surgery, before the West Virginia Medical Association, at Elkins, October, 1909.

